# Effects of bacterial wilt on community composition and diversity of culturable endophytic fungi in *Alpinia galanga*

**DOI:** 10.3389/fmicb.2025.1536079

**Published:** 2025-07-16

**Authors:** Jiahui Liu, Yuanyuan Wu, Jinru Lin, Mengxia Xie, Likai Chen, Liguo Wang

**Affiliations:** ^1^Laboratory of Germplasm Resources and Molecular Identification of Traditional Chinese Medicine, School of Pharmaceutical Sciences, Guangzhou University of Chinese Medicine, Guangzhou, China; ^2^Artemisinin Research Center, Guangzhou University of Chinese Medicine, Guangzhou, China; ^3^Key Laboratory of Lingnan Chinese Medicine Resources, Ministry of Education, Guangzhou University of Chinese Medicine, Guangzhou, China

**Keywords:** *Alpinia galanga*, endophytic fungi, community composition, diversity, abundance

## Abstract

*Alpinia galanga* Willd is a perennial herbaceous plant that usually has a stable microflora living in the inter-root and stem and leaf tissues, which assists the host in normal growth and development. The bacterial wilt disease investigated in *A. galanga* planting bases is a novel soil-borne disease caused by the pathogenic bacterium *Ralstonia solanacearum* (Smith) Yabuuchi et al., which disrupts the *A. galanga*-microbe-soil microecological balance. For this reason, it is important to study the changes in endophytic fungal community growth and diversity in healthy and diseased *A. galanga*, and to mine the active endophytic fungal resources in order to lay the foundation for exploring the functional microbial communities for artificial synthesis. From 685 endophytic fungi strains isolated from healthy (HDK_J) and diseased (HDK_B) *A. galanga* stems/leaves, 27 species were identified in HDK_J and 8 in HDK_B (belonging to 3 Phyla, 6 Classes, 13 Families). HDK_B’s fungal relative abundance (RA) was only 38.93% of HDK J’s, indicating significantly lower composition/abundance. While species in stems and leaves were identical within health groups, leaf RA exceeded stems by 124.23% in HDK_J and 78.23% in HDK_B. The RA of HDK_J leaves was 78.08% higher than that of stems. All diversity indices for HDK_J were higher than those for HDK_B, with significant differences. The phylogenetic trees revealed four major branches of endophytic fungi species in HDK_J, and especially, there were many long development branches under the Ascomycota. In contrast, the phylogenetic tree for HDK_B showed only one major branch (Ascomycota) with few sub-branches. The bacterial wilt significantly affected the composition and RA of endophytic fungi in *A. galanga*. The diversity indices showed a decreasing trend in *A. galanga* after being infected by *R. solanacearum*. The dominant species were changed. The parts of sensitive endophytic fungi had disappeared. This result will be helpful for studies on the relationship between the artificial minimal microbial community and the role of the host, as well as for studies on synthetic microbiomics.

## 1 Introduction

Plant growth is susceptible to various biotic and abiotic stress factors, and the normal development process of plants is impeded under the stress, which affects the traits and quality of plants. The stressed plants are not only caused by the pests and diseases, but also by abiotic factors such as drought, waterlogging, soil salinity, air pollution, excessive heavy metals, and nutrient deficiency. Stressed plants trigger a series of responses in the host, including changes in various physiological and biochemical indices in metabolic pathways and adjustments in the microenvironment of the host tissue system in which the microbes live (Ashraf and Harris, 2013). There are a lot of endophytes living in plant tissues, and their community structure is closely related to the hosts and the ecological environments. When plants live in a specific geographical environment for a long time, there are often stable endophyte community systems in the plant tissues. The endophytic flora co-exist and interact with the host for a long period of time. The small molecule metabolites produced by either the host or the endophytes during the interaction play a key role in regulating the growth and development of the host, the defense of the system, and the synthesis and accumulation of the secondary metabolites (Oldroyd and Downie, 2008; Moghaddam et al., 2021; Verma et al., 2021). After healthy plants are infected with diseases, the balanced microenvironment of the hosts is disrupted *in vivo*, leading to changes in the endophyte community and the abundance of the endophyte constitution. Some inferior endophytic flora become dominant, while others disappear, and some endophytes become dominant endophytes, which affect the functions of the endophyte community (Lin et al., 2022). An increasing number of studies on endophytes have shown the diversity of the endophytes functions, which are not identical for different active endophytes. Competitive endophytes have the ability to compete with pathogens for nutrients, occupy invasion sites, and resist the invasion of pathogens, while antagonistic endophytes inhibit the expansion and spread of pathogens in host tissues (Sornmakili et al., 2020). With the fluctuation of the endophyte community of the hosts, the normal interspecific coexistence, mutuality, symbiosis, parasitism, competition, and antagonism between the endophyte community and the exogenous pathogenic microorganisms are changed, especially the changes of the endophyte community composition with competitive or antagonistic ability will affect the development of the host diseases. More studies have found that the community structure of endophytes is related to disease resistance, pest resistance, and stress resistance in hosts (Kamran et al., 2022; Ma et al., 2023; Yang et al., 2024). By studying the changes in the structural composition and diversity of endophyte communities in healthy and infected hosts, a foundation can be laid for exploring the construction of recombinant artificial endophytes and their functions.

Hongdoukou is a perennial herb in the ginger family (Zingiberaceae), and its mature dried fruits and rhizomes are two different Chinese medicinal materials, which are commonly used as medicinal and food herbs cultivated in Guangdong, Guangxi, and Yunnan Provinces in China. In recent years, bacterial wilt has become a serious disease threatening the growth of *A. galanga*. Our research team first identified the causative agent causing bacterial wilt according to Koch’s rule, which expands the host range of pathogenic bacteria. Bacterial wilt is a bacterial soilborne disease caused by *Ralstonia solanacearum*, which can cause damage in many tropical and subtropical crops. Bacterial wilt is a typical vascular disease, in which *R. solanacearum* invades the host from the root wounds, passes through the cortex into the vascular bundles, and spreads upward along the vascular bundles, during which the mass bacterial propagules and secretions adhere to the inner wall of the conduit to form a biofilm barrier to block the conduit, causing leaves to lose water and green withering (Xue et al., 2020). Earlier, the studies of bacterial wilt mainly focused on the interacting mechanism between the pathogenic process of *R. solanacearum* and host resistance. It was detected that *R. solanacearum* secretes extracellular polysaccharides (EPS) (Perraud et al., 2017) and a variety of pathogenicity-associated effector proteins (EPs) (Remigi et al., 2011), which are virulence factors that determine the pathogenicity and host range of *R. solanacearum* (Genin, 2010; Fabienne and Stéphane, 2023). Meanwhile, the signaling channels of the innate immune system of the host have also been studied from the perspective of host-pathogen interaction, and quite a few of signaling components and cell membrane surface pattern-recognition receptors (PRRs) have been discovered and examined (Wang et al., 2020; Manhães et al., 2021). In recent years, with the development of multi-omics analysis techniques, the rhizobial microbiome and endophytic flora that can help the host to resist bacterial wilt have been increasingly reported. A number of useful bacteria and fungi have been found, indicating that the plant immune system and microbiomes interact synergistically with each other to defend against *R. solanacearum* (Agarwal et al., 2020; Cai et al., 2021). In our previous study on the community structure of endophytic fungi, we found that their stability was greatly affected by the external environment. It was hypothesized that the changes in the host tissue microenvironment would affect the endophytic fungal community composition and diversity before and after *A. galanga* is infested by *R. solanacearum*. Therefore, we have isolated endophytic fungi from both healthy and diseased materials of *A. galanga* with the traditional fungal PDA culture method to study the differences in the composition of the culturable endophytic fungal community and the changes in diversity in the two materials. Furthermore, it aims to explore the synergistic role of endophytic fungi and the host immune system in defense against bacterial wilt.

## 2 Materials and methods

### 2.1 Source of materials

**Figure 1:**
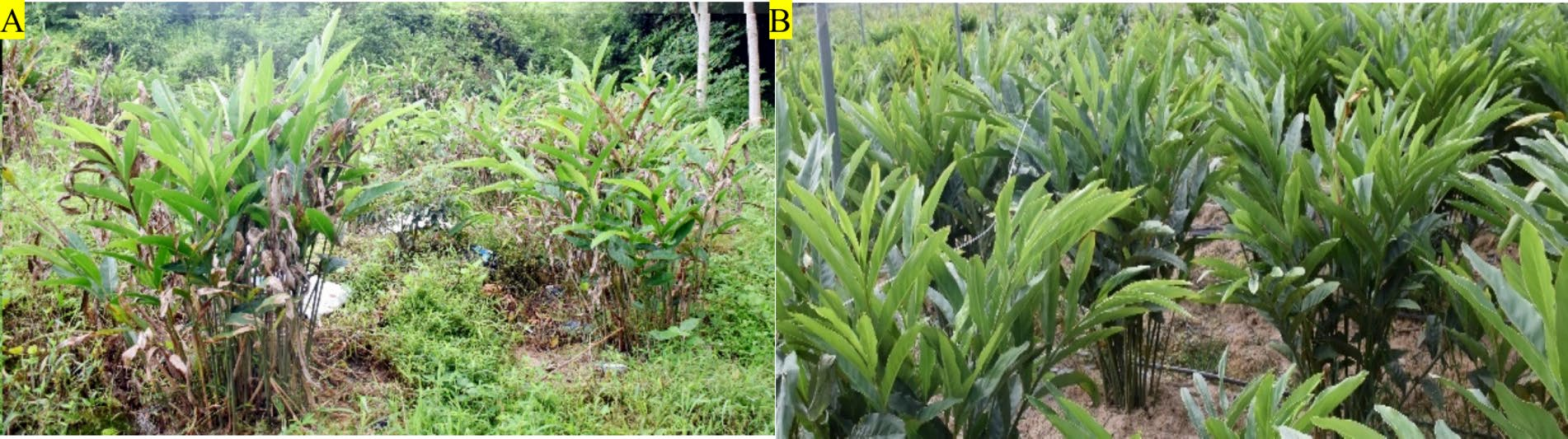
The growth of healthy and diseased plants of *A. galanga* in the planting bases. **(A)** HDK_B; **(B)** HDK_J.

From March 2022 to May 2023, diseased samples of *A. galanga* (No. HDK_B) were collected from the growing regions that are managed by an agricultural company in Chaozhou City in China (Figure 1A). According to the epidemiological characteristics of soilborne diseases, healthy *A. galanga* (No. HDK_J) were collected from distant mountainous regions far away from the pathogen-contaminated soils in Chaozhou City, China’s Guangdong Province. (Figure 1B). Healthy and diseased samples were collected in batches at three growth stages, approximately 5 months apart, at which time the new bacterial wilt spreads from plant to plant. One branch was cut from each plant cluster, and five branches were collected from each healthy and diseased *A. galanga*. After all samples were taken back to the laboratory, the stems (No. HDK_S) and leaves (No. HDK_L) of the whole branches were separated, rinsed with sterile water, and dried. Then, all stems and leaves were clipped to 10 cm, packaged in sealing bags, and stored in a refrigerator at 4°C for later use.

### 2.2 Incidence investigation

The surveyed planting plots of *A. galanga* were the same as those of the collected samples, and the investigation time was during severe periods of bacterial wilt in May 2023. The incidence (%) and the severity of bacterial wilt were investigated, and the disease index (DI) was calculated. Incidence (%) = the number of infected plant clusters/the total number of investigated clusters × 100. The classification criteria for the severity of bacterial wilt of *A. galanga* refer to the national standards for the classification of tobacco bacterial wilt disease in China (GB/T 23224–2008) (National Standard of the People’s Republic of China, 2008). The severities of bacterial wilt are divided as follows: Grade 0: no diseases on the whole plant; Grade I: the scattered lesions at the bases of the stems, and narrow and discontinuous lesions, and no wilting of leaves; Grade III: the lesion fusion at the bases of the stems, and not more than 1/3 lesions around the stem circumference, and 1/3 lightly wilting leaves, and a few green withering in leaves of the lower parts of the plants; Grade V: more than 1/3 areas of merging lesions around the stem circumference, and 1/2 green withering leaves; Grade VII: all lesions around the stem circumference, and more than 2/3 green withering leaves; Grade IX: green withering and dead in all leaves. Disease index (%) = ∑ (the number of diseased plant clusters at all levels × severity at all levels) / (total number of investigated plant clusters × highest severity) × 100[GB / T-23224-2008] (National Standard of the People’s Republic of China, 2008).

### 2.3 Material handling

The samples were processed in three batches, and five healthy and diseased stems and leaves were randomly selected in each sample. The stems were cut into 5 cm lengths, and the leaves were cut into 5 × 5 cm sizes. The order of sterilization is as follows: the stems and leaves were placed in beakers containing 75% ethanol for 3 min, and then rinsed three times in sterile water. Subsequently, the materials were soaked in 0.1% mercuric acid, and Tween-20 was added for 1.5 min for stems and 2 min for leaves. During the immersion period, the stems and leaves were stirred continuously so that they were in full contact with the sterilizing solution, and were removed and rinsed with sterile water for 1 min each time for 3 times, finally, the samples were blotted dry with sterile filter papers for later (Sahu et al., 2022; Xiang et al., 2018).

### 2.4 Isolation, culture, purification, and preservation of strains

A total of 60 tissue blocks were cut from healthy and diseased material under aseptic conditions by cutting the leaves into 1.0 cm × 1.0 cm size and stems into 0.5 cm size. Every three tissue blocks were placed on PDA, and the tissue blocks were rolled or laid on the surface of PDA for 2 min in advance to avoid microbial contamination as a control. Endophytic fungi were cultured in healthy tissue blocks with PDA medium. PDA and TTC (Guangdong Hankai Microbial Technology Co., Ltd., China) medium were used for the isolation and culture of endophytic fungi and pathogenic bacteria in diseased tissue blocks. The petri dishes were incubated at 25°C under constant temperature and humidity and observed on the next day. When the mycelium grew on the cut surface of the tissue block, it was picked out from the edge for isolation. Strain purification was performed by single picking of hyphae at the edge of the colony in the early stage, and single spore isolation was used after spore production. The purified strains were numbered and inoculated into 9 cm test tubes and stored under refrigeration at 2 ~ 4°C for strain classification and identification (Gagne-Bourgue et al., 2013; Terna et al., 2024).

### 2.5 Strain identification

#### 2.5.1 Morphological identification

After the activation of the preserved strains, morphological observation and preliminary screening were carried out using a ZEISS (Axio Scope) optical microscope. The identification contents mainly included colony morphology, hypha growth characteristics, conidial stalk and conidiospore morphology, sporulation structure, and mode of sporulation. Strains exhibiting low sporulation efficiency were cultured for sporulation and then identified after sporulation according to the above methods (Domsch et al., 1980; Sun W. et al., 2023).

#### 2.5.2 Molecular identification

The initial screened strains were identified using the fungal ITS amplicons as a molecular marker, and the DNA was extracted using the fungal genome DNA extraction kit (Beijing Soleberg Technology Co., Ltd., China). The primers used for PCR amplification were ITS1 (5’-TCCGTAGGTGAACCTGCGG-3′) and ITS4 (5’-TCCTCCGCTTATTGATATGC-3′) (Beijing Soleberg Technology Co., Ltd., China) (Ebadi et al., 2024; Sarsaiya et al., 2020), and the DNA ITS1/ITS4 sequences were amplified. PCR amplification systems (50 μL): DNA template (100 ng.μL^−1^) 1 μL, primers ITS1 and ITS4 2 μL of each, gold mix (green) 45 μL. The PCR amplification procedure included: pre-denaturation at 98°C for 2 min, denaturation at 98°C for 10 s, annealing at 55°C for 10 s, extension at 72°C for 90 s, a total of 35 cycles, and finally extended at 72°C for 1 min, and the reaction was terminated at 4°C (Yang et al., 2023; Lima et al., 2012). According to the ratio of adding 1 μL of DNA sample loading buffer to 5 μL of DNA sample, the two were mixed for later use. The mixture was directly loaded for sampling with the DL2000 DNA marker, and the samples were spotted onto 1% agar gel. The PCR products were visualized on a 1% agarose gel stained with ethidium bromide. Electrophoresis was conducted under the following parameters: 100 V voltage, 500 mA current, 250 W power, and a run time of 35–40 min. The amplified products were sequenced by the Guangzhou Company, Shenzhen BGI Genomics Institute, China.

The measured ITS sequences were identified using the fungal Database (UNITE Database) (Nilsson et al., 2019), and the sequences with no or ambiguous matches in the identification process were then compared by BLAST in NCBI’s GenBank and were downloaded (Ouziad et al., 2005; Schoch et al., 2012; Jiang et al., 2016). Clustal X software (version 1.83) was used for multiple sequence comparisons, and MEGA software (version 11.0) was used for the identification of relatedness and the construction of a phylogenetic tree by the maximum-likelihood (ML) method (Chen et al., 2013; Sun W. et al., 2023).

### 2.6 Data processing and calculations

The analysis of diversity indicators included: (1) Colonization rate (CR) reflects the abundance of endophytic fungi in the host, and CR (%) = (the numbers of tissue blocks isolated from which endophytes were isolated / the numbers of total tissue blocks) × 100; (2) Relative separation frequency (SF) reflects the occurrence frequency of a microbial species in the endophytic fungal community in the same material, and SF (%) = (the numbers of isolated microbial species / the total numbers of all isolated microbial species) × 100; (3) Relative abundance (RA) refers to the proportion of the numbers of individual species in the colony to the total numbers of species in the community; (4) Shannon-Wiener index (*H′*) is used to compare and to analyze the species richness level of endophytic fungal communities between healthy and diseased materials, and the calculation formula as follows: *H′=*$\sum_{i=1}^{k}P$×In *P*_i_, where *P_i_* refers to a species percentage in all species, and higher values represent higher community diversity and complexity (Shannon, 1948). (5) Simpson diversity index (*D*) was used to assess endophytic fungal community diversity, and the greater the values are, the greater the community diversity is. The calculation formula as follows: *D* = 1-Σ(*P*_i_)^2^, and where *P*_i_ represents the proportion of numbers of species in all species (Simpson, 1949); (6) Margalef richness index (*R*) is calculated as follows: *R* = (S-1) / ln *N*, and where S represents the microbial numbers of species, and N represents the total numbers of samples; (7) Pielou evenness index (*J*) is calculated by the formula: *J* = *H′*/ln S, and where *H′* is Shannon-Wiener index, and *S* represents the numbers of species. Diversity indices among different samples were analyzed for significance of difference using a one-way ANOVA test (*p* < 0.05), and the homogeneity of variance was tested by Levene’s test. When *p* < 0.05, the difference was significant. All statistical analyses were performed using Excel software and biostatistical software SPSS.

The difference analysis of community composition of endophytes includes: After molecular identification, the relative abundance of endophytic fungi species was analyzed at the genus and species level. Excel comma-separated value files (.csv) and ggplot2 software were used to draw the clustering heat maps and relative abundance stack maps of endophytic fungi species using the R language (Zhang J. Z. et al., 2024; Jin et al., 2023).

## 3 Results and analysis

### 3.1 Field investigation of bacterial wilt

**Figure 2:**
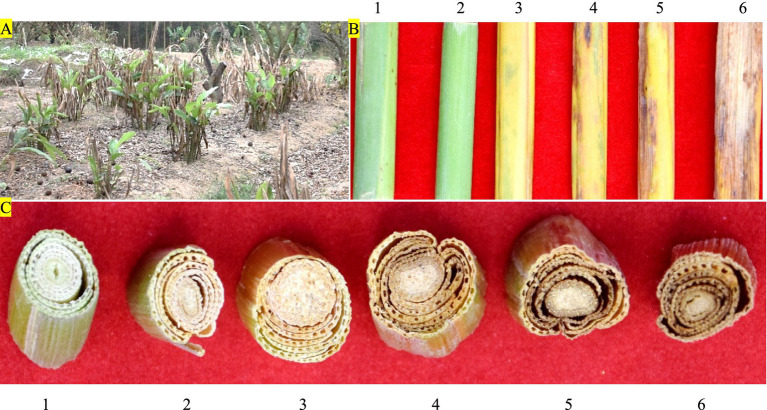
The incidence and severity of bacterial wilt in HDK_B. **(A)** The incidence of bacterial wilt in HDK_B. **(B)** The severity of bacterial wilt in the stems of HDK_B. **(C)** The severity of bacterial wilt at the basal parts of the stem transections in HDK_B. 1: HDK_J. 2: the severities at level I in HDK_B. 3: the severities at level III in HDK_B. 4: the severities at level V in HDK_B. 5: the severities at level VII in HDK_B. 6: the severities at level IX in HDK_B.

*Alpinia galanga* rhizome is stout and well developed with a bright red surface, and the mother buds develop to form plant clusters ranging from 8 to 15 branches above ground per cluster. Taking the plant cluster as the unit of investigation, the average incidence of bacterial wilt was 92.24% (Figure 2A). The severity of aboveground branches varied from cluster to cluster, with older shoots having severe diseases and newer shoots having less or no disease. The highest disease index (DI) in the aboveground branches of infected plant clusters was 87.11%, and the average DI of all investigated plant clusters was 73.42%. The proportion of branch severities grade 0 is 6.32%, grade I 8.24%, grade III 17.55%, grade V 27.23%, grade VII 26.41%, and grade IX 14.25%. (Figures 2B,C).

### 3.2 Community structure of culturable endophytic fungi

Samples of healthy and diseased *A. galanga* were collected at different growth stages, and endophytic fungi were isolated and identified in 3 batches per stage. A total of 685 endophytic fungal strains were isolated, and a total of 556 strains were genetically characterized by fungal ITS genes after morphological characterization of colonies, hyphae, and conidia. Twenty-seven species of endophytic fungi were identified by ITS sequence alignment in the UNITE database and by BLAST software in the GenBank database, excluding duplicate species, of which 27 species were isolated from the healthy stems and leaves of *A. galanga*, and eight species were isolated from diseased *A. galanga*. The 27 species are: *Alternaria* sp., *Epicoccum nigrum*, *E. andropogonis*, *E.* sp., *Pezicula* sp., *Colletotrichum boninense*, *C. gloeosporioides*, *C. fructicola*, *C.* sp., *Diaporthe subclavate*, *D.* sp., *Physalospora* sp., *Pestalotiopsis microspore*, *Nigrospora oryzae*, *N. musae*, *N.* sp., *Arthrinium arundinis*, *A.* sp., *Fusarium proliferatum*, *F. oxysporum*, *F.* sp., *Mycoleptodiscus indicus*, *M.* sp., *Xenoacremonium recifei*, *Trichoderma* sp., *Schizophyllum commune* and *Mucor fragilis*. Among them, the species of *Colletotrichum* were most abundant in healthy and diseased stems and leaves of *A. galanga*. They had the highest isolation frequency, indicating that the species of *Colletotrichum* are dominant endophytes. According to the latest mycological classification system, 27 species were categorized into 3 Phyla, 6 Classes, 9 Orders, 13 Families, and 16 Genera, of which 16 were known species and 11 were unknown species (Table 1).

**Table 1:** The composition of the community structure of endophytic fungi in the *A. galanga* plant.

Phylum	Class	Order	Family	Genus	Species
Ascomycota	Dothideomycetes	Pleosporales	Pleosporaceae	*Alternaria*	*A.* sp.
				*Epicoccum*	*E. nigrum*
					*E. andropogonis*
					*E.* sp.
	Leotiomycetes	Helotiales	Dermateaceae	*Pezicula*	*P.* sp.
	Sordariomycetes	Trichosphaeriales	Glomerellaceae	*Colletotrichum*	*C. boninense*
					*C. gloeosporioides*
					*C. fructicola*
					*C.* sp.
		Diaporthales	Diaporthaceae	*Diaporthe*	*D. subclavata*
					*D.* sp.
			Hyponectriaceae	*Physalospora*	*P.* sp.
			Melanconidaceae	*Pestalotiopsis*	*P. microspora*
				*Nigrospora*	*N. oryzae*
					*N. musae*
					*N.* sp.
		Xylariales	Apiosporaceae	*Arthrinium*	*A. arundinis*
					*A.* sp.
		Hypocreales	Nectriaceae	*Fusarium*	*F. proliferatum*
					*F. oxysporum*
					*F.* sp.
			Hypocreaceae	*Mycoleptodiscus*	*M. indicus*
					*M.* sp.
			Clavicipitaceae	*Xenoacremonium*	*X. recifei*
	Xylonomycetes	Xylonomycetales	Xylonomycetaceae	*Trichoderma*	*T.* sp.
Basidiomycota	Agaricomycetes	Agaricales	Schizophyllaceae	*Schizophyllum*	*S. commune*
Zygomycota	Zygomycetes	Mucorales	Mucoraceae	*Mucor*	*M. fragilis*

Further analysis of the taxonomic status of the 27 species showed that these species belonged to three phyla: Ascomycota, Basidiomycota, and Zygomycota, with Ascomycota being the dominant phylum, accounting for 92.60%, followed by 3.70% for both Ascomycota and Zygomycota. At the class level, 27 species belonged to six classes, including four classes of Ascomycota, namely Dothideomycetes, Leotiomycetes, Sordariomycetes, and Xylonomycetes, and one class of Basidiomycota, Agaricomycetes, and one class of Zygomycota, Zygomycetes. According to the included species numbers, the order of classes was Sordariomycetes (nineteen species) > Dothideomycetes (four species) > Leotiomycetes, Xylomycetes, Agaricomycetes, and Zygomycetes (one species), of which Sordariomycetes was the dominant class, accounting for 70.37% of the total number of species. At the order level, 27 species belonged to nine orders, including seven orders of Ascomycota, namely Pleosporales, Helotiales, Trichosphaeriales, Diaporthales, Xylariales, Hypocreales, and Xylonomycetales, Basidiomycota one, Agaricales, Zygomycota one, and Mucorales. In order of the species included, the ranking order was Diaporthales (seven species) > Hypocreales (six species) > Pleosporales and Trichosphaeriales (four species) > Xylariales (two species) > Helotiales, Xylonomycetales, Agaricales, and Mucorales (one species), of which Diaporthales was the dominant order, accounting for 25.93% of the total species. At the family level, 27 species belonged to 13 families, including 11 families of Ascomycota, namely Pleosporaceae, Dermateaceae, Glomerellaceae, Diaporthaceae, Hyponectriaceae, Melanconidaceae, Apiosporaceae, Nectriaceae, Hypocreaceae, Clavicipitaceae, and Xylonomycetaceae, and one family of Basidiomycota, Schizophyllaceae, and one family of Zygomycota, Mucoraceae. Among them, Pleosporaceae (four species), Glomerellaceae (four species), and Melanconidaceae (four species) were the dominant families, accounting for 44.44% of the total species (Table 1).

### 3.3 Analysis of endophytic fungal diversity index

**Figure 3:**
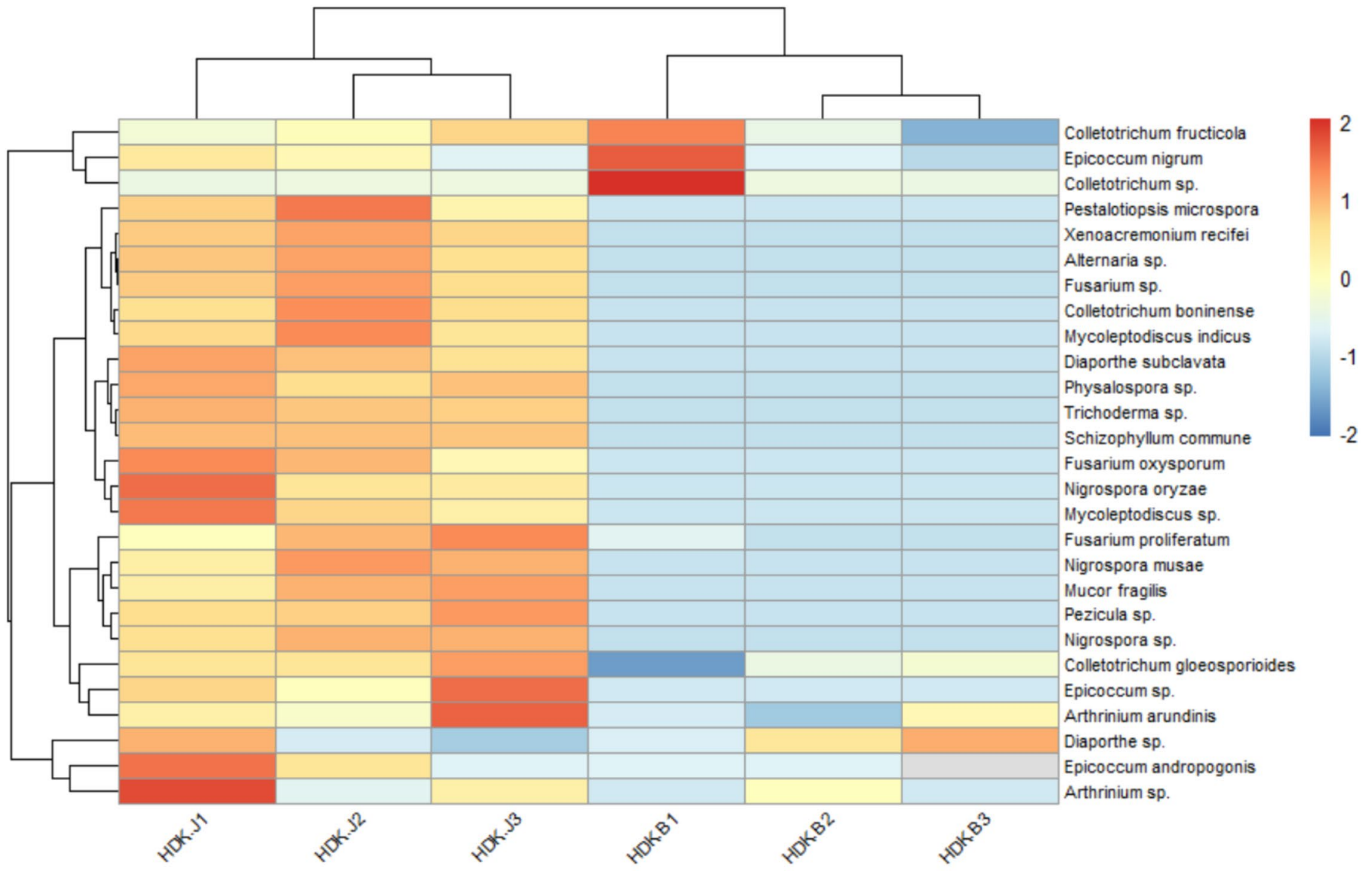
Abundance clustering heat map of endophytic fungi in HDK_J and HDK_B at the species level. HDK_J1, HDK_J2 and HDK_J3: three samples of HDK_J. HDK_B1, HDK_B2 and HDK_B3: three samples of HDK_B.

The colonization and community composition of endophytic fungi were more related to the parts and health status of *A. galanga*. The colonization rate (CR) and diversity index (DI) of endophytic fungi in leaf tissues were higher than those in stems, and the CR and DI of endophytic fungi were higher in healthy *A. galanga* than those in diseased *A. galanga*, which were significantly different (*p* < 0.05). As can be seen from Table 2: The leaf endophyte colonization rate was 130.94% of that of the stems, and the Shannon-Wiener index (*H′*), Simpson’s index (*D*) and Margalef’ s index (*R*) were HDK_J leaves (1.16, 0.87, 5.12) > HDK_J stems (0.82, 0.72, 4.22) > HDK_B leaves (0.57, 0.24, 2.97) > HDK_B stems (0.33, 0.14, 2.25). These results showed that bacterial wilt disease had a great impact on the microenvironment of *A. galanga* stem and leaf tissues, destroying endophytic fungal diversity and the stability of community structure. Comparatively, the effect on the stem microenvironment was greater than that on the leaves. The relative separation frequency (SF) of dominant endophytic species in the same batch of tissue blocks from healthy and diseased *A. galanga* was that HDK_J (26.46%) was lower than HDK_B (50.69%), which was significantly different (*p* < 0.05). The results indicated that the decrease in the relative abundance (RA) of endophytic fungal species in HDK_B caused the SF fluctuations of endophytic fungi in host tissues, and the SF was raised in HDK_B. At the same time, the Pielou index (*J*) of HDK_J leaves was higher than that of HDK_J stems and HDK_B stems and leaves, and the uniformity of HDK_J stems and HDK_B stems and leaves was not much different, indicating that the endophytic fungi species in HDK_J leaves were more evenly distributed (Table 2). The relative abundance clustering heat map of species showed that the community structure and composition of the endophytic fungi in the three groups of samples (HDK_J1, HDK_J2, and HDK_J3) from HDK_J were relatively stable, with little change in relative abundance of the 27 species, whereas the structure and composition in the three groups of samples (HDK_B1, HDK_B2, and HDK_B3) from HDK_B fluctuated greatly, with some of the stress-sensitive species disappearing, and some of the species turning out to be dominant endophytic fungi (Figure 3).

**Table 2:** The diversity index of the community structure of endophytic fungi in HDK_J and HDK_B.

Incidence	Organs	Colonization rates (%)	SF of dominant genera (%)	Shannon-Wiener index (*H′*)	Simpson index (*D*)	Margalef index (*R*)	Pielou index (*J*)
Healthy samples	Stem	44.24 ± 1.02 ^b^	18.14 ± 0.76 ^a^	0.82 ± 0.06 ^a^	0.72 ± 0.03 ^ab^	4.22 ± 0.08 ^b^	0.50 ± 0.09 ^a^
	Leaf	69.45 ± 0.76 ^a^	34.78 ± 1.13 ^a^	1.16 ± 0.14 ^a^	0.87 ± 0.11 ^a^	5.12 ± 0.16 ^a^	0.71 ± 0.02 ^a^
Diseased samples	Stem	16.78 ± 0.61 ^d^	30.97 ± 0.98 ^b^	0.33 ± 0.47 ^b^	0.14 ± 0.06 ^c^	2.25 ± 0.04 ^c^	0.48 ± 0.12 ^ab^
	Leaf	32.45 ± 0.47 ^bc^	70.41 ± 0.26 ^c^	0.57 ± 0.65 ^b^	0.24 ± 0.04 ^b^	2.97 ± 0.07 ^c^	0.54 ± 0.17 ^b^

Data are means ± SD (*n* = 3). Different lowercase letters in the same column show significant differences of the mean by LSD test (*p* < 0.05); SF of dominant genera: Relative separation frequency of dominant genera.

### 3.4 Differences in the community composition in healthy and Diseased *Alpinia galanga*

**Figure 4:**
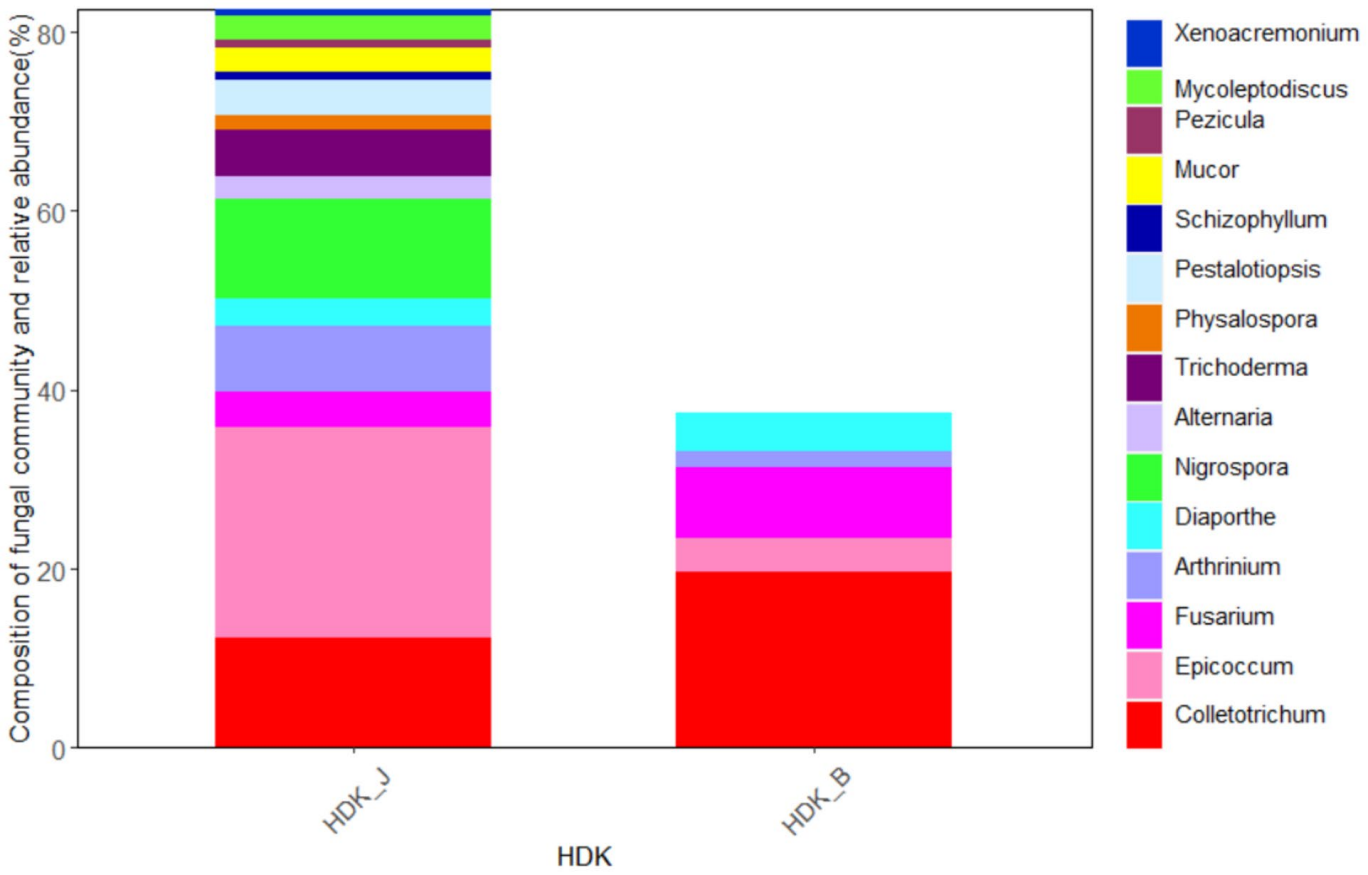
The community composition and relative abundance of endophytic fungi in HDK_J and HDK_B at the genus level.

At the genus level, the endophytic fungi isolated from HDK_J belonged to 15 genera, including 13 genera in the *Ascomycota*, namely, *Alternaria*, *Epicoccum*, *Pezicula*, *Colletotrichum*, *Diaporthe*, *Physalospora*, *Pestalotiopsis*, *Nigrospora*, *Arthrinium*, *Fusarium*, *Mycoleptodiscus*, *Xenoacremonium*, and *Trichoderma*, a genus in the *Basidiomycota*, *Schizophyllum*, and a genus in the Zygomycota, *Mucor*. The dominant genus was *Epicoccum* at the genus level with a relative abundance of 36.46%. Meanwhile, HDK_B was divided into five genera, namely, *Epicoccum*, *Colletotrichum*, *Diaporthe*, *Arthrinium*, and *Fusarium*, all of which were endophytic fungi of the *Ascomycota*. The dominant genus in HDK_B was *Colletotrichum*, with a relative abundance of 50.69%. Comparing the relative abundance of the HDK_J and HDK_B at the genus level, HDK_B endophytic fungi were only 38.93% of HDK_J. (Figure 4).

**Figure 5:**
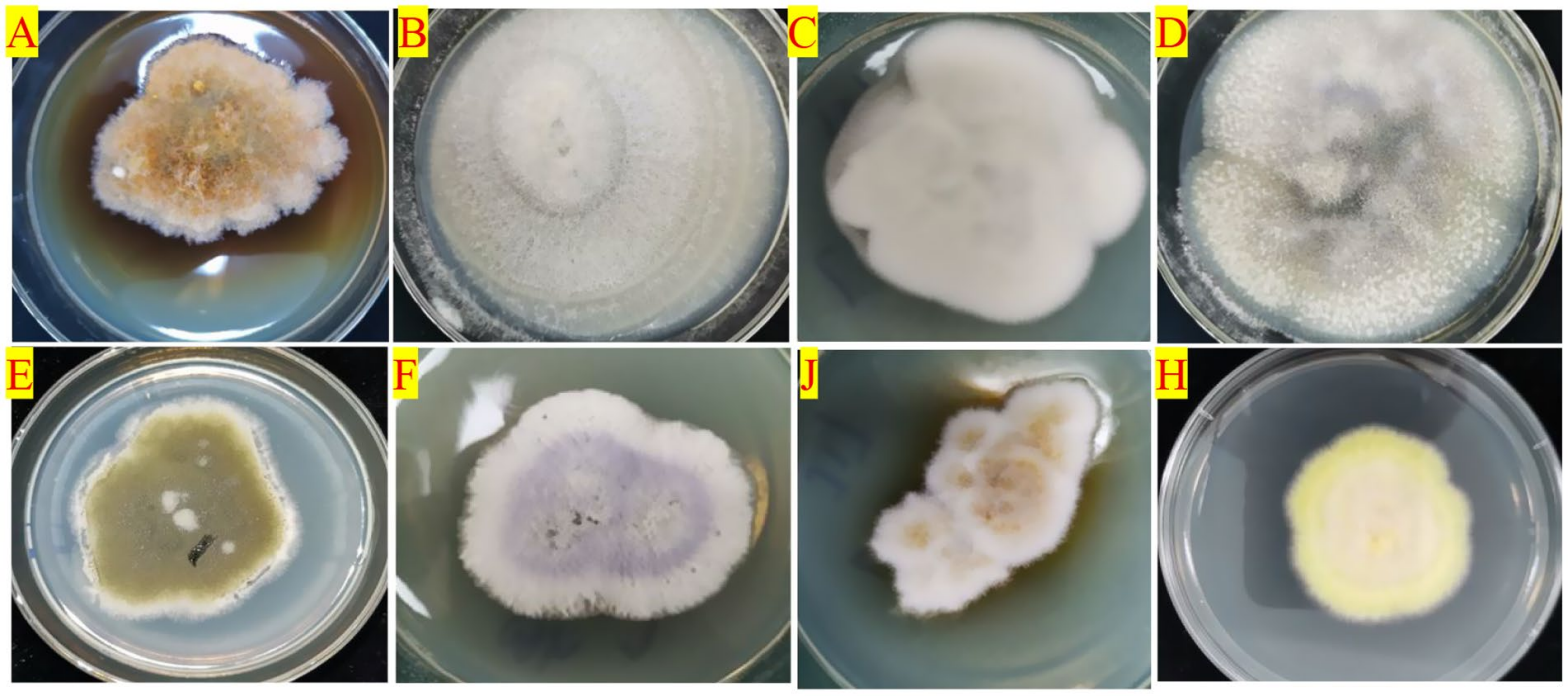
The colony morphology of the eight species of endophytic fungi producing pigments isolated from HDK_J/HDK_B cultured on PDA medium. **(A)** *Epicoccum nigrum*, **(B)** *Colletotrichum gloeosporioides*, **(C)** *C. fructicola*, **(D)** *C*. sp., **(E)** *Arthrinium arundinis*, **(F)** *Fusarium proliferatum*, **(G)** *A*. sp., **(H)** *Diaporthe* sp.

**Figure 6:**
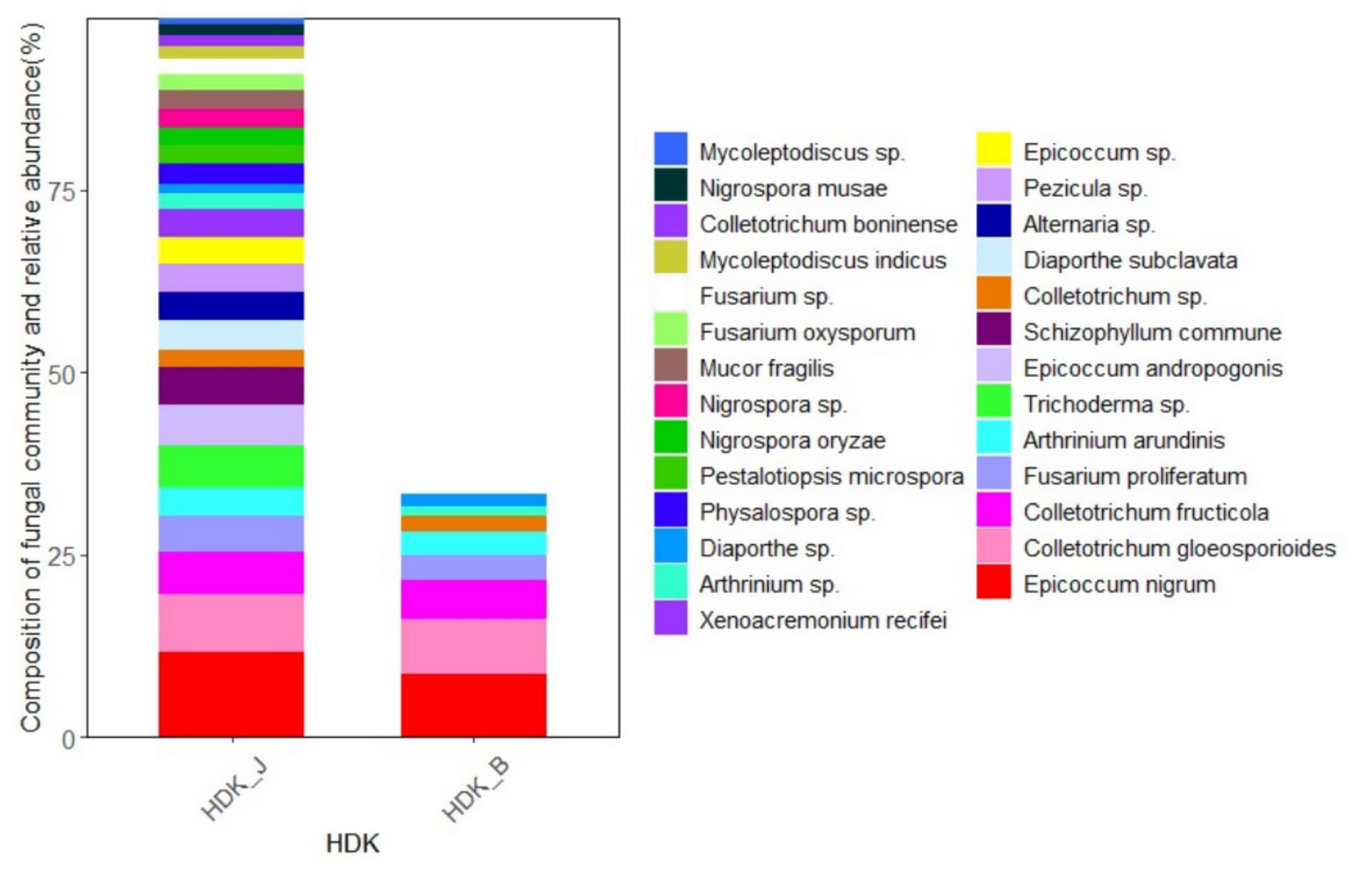
The community composition and relative abundance of endophytic fungi in HDK_J and HDK_B at the species level.

At the species level, the endophytic fungi species of HDK_J and HDK_B were significantly different. Among the 27 isolated endophytic fungi, HDK_J and HDK_B shared eight species, five of which are known, namely, *E. nigrum*, *C. gloeosporioides*, *C. fructicola*, *A. arundinis*, and *F. proliferatum*. Three unknown species, namely, *Colletotrichum* sp., *Diaporthe* sp., and *Arthrinium* sp. These eight shared endophytic fungi had a strong pigment-producing ability, among which *E. nigrum* belonged to *Leotiomycetes*, and the others belonged to *Sordariomycetes*. Most of the eight endophytic fungi were semi-living trophic fungi with a low degree of specialization, and some of them were potentially pathogenic. The metabolism of the eight fungi in culture was vigorous, and the ability to produce secondary metabolites was strong, especially pigmented substances (Figure 5). On the other hand, the 19 endophytic fungi isolated from HDK_J, including *Alternaria* sp., *E. andropogonis*, *Epicoccum* sp., *Pezicula* sp., *Colletotrichum boninense*, Diaporthe subclavata, *Physalospora* sp., *Pestalotiopsis microspora*, *Nigrospora oryzae*, *N. musae*, *N.* sp., *Fusarium oxysporum*, *F.* sp., *Mycoleptodiscus indicus*, *M.* sp., *Xenoacremonium recifei*, *Trichoderma* sp., *Schizophyllum commune* and *Mucor fragilis*, were sensitive to the stress of bacterial wilt, which affected the stability of endophytic fungal community structure in *A. galanga* (Figure 6).

### 3.5 Differences in the community composition of the stems and leaves of *Alpinia galanga*

**Figure 7:**
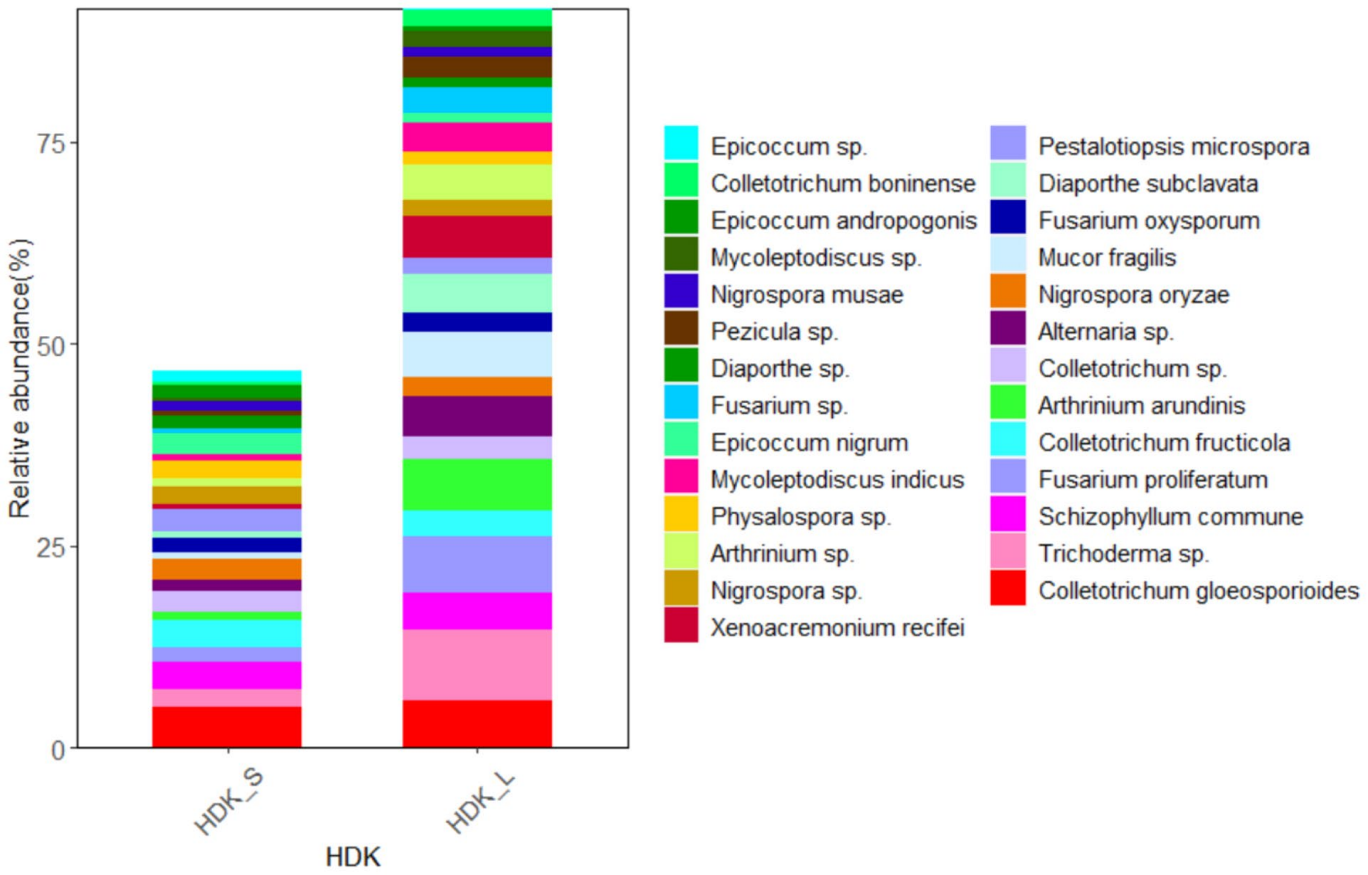
The composition of community and relative abundance of endophytic fungi in the healthy HDK_S and HDK_L.

**Figure 8:**
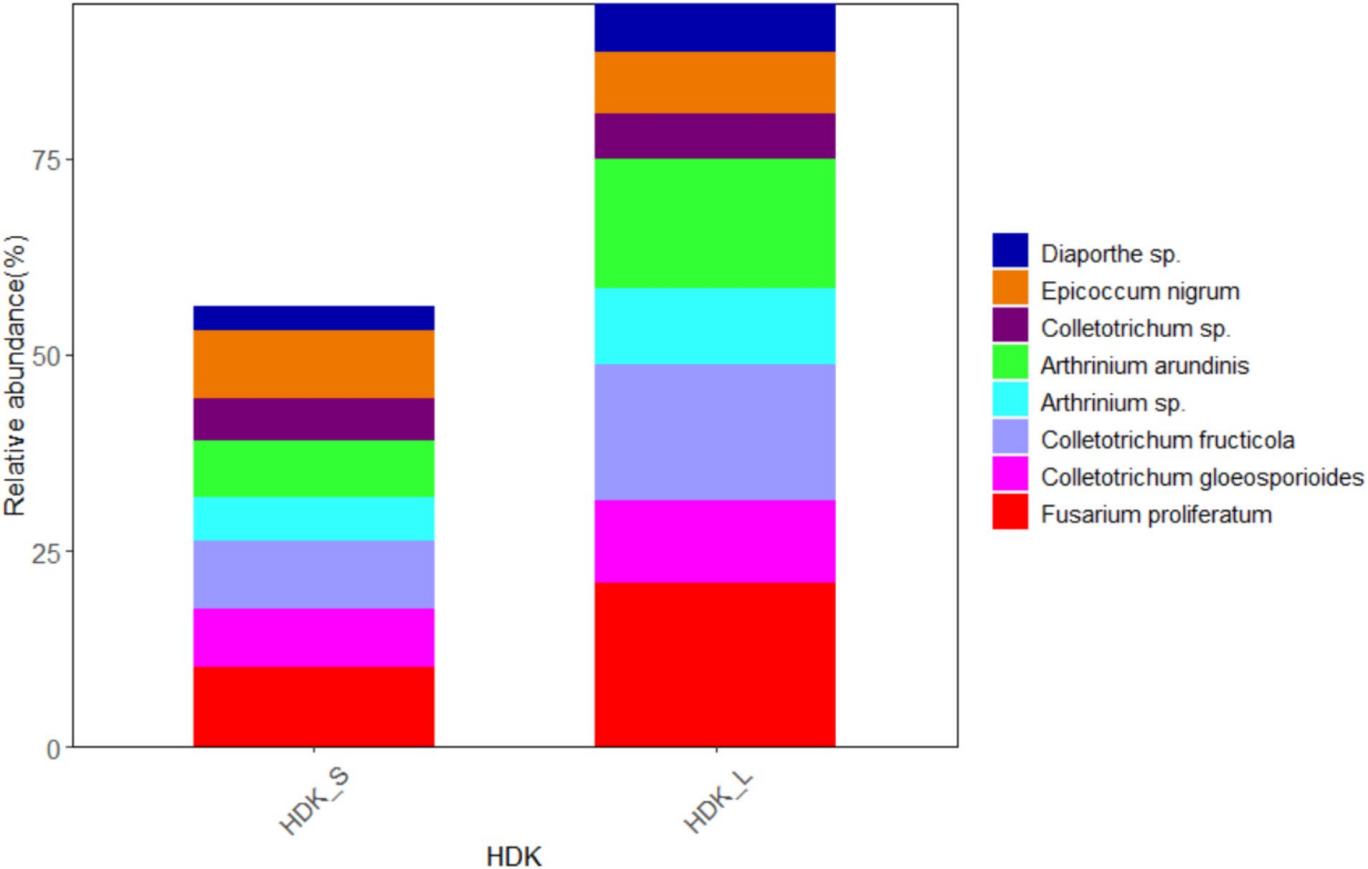
The composition of community and relative abundance of endophytic fungi in the diseased HDK_S and HDK_L.

The results of isolated endophytic fungi from the stems and leaves of *A. galanga* showed that both organs had abundant endophytic fungi, but the relative abundance of leaves was higher than that of stems. Twenty-seven endophytic fungi were isolated from the stems and leaves of HDK_J, and the number of isolated species was the same, but the relative abundance of leaf organs was 124.23% higher than that of stem organs (Figure 7). Eight endophytic fungi were isolated from stems and leaves of HDK_B, with the same number of species in stems and leaves, but the relative abundance of leaf organs was 78.08% higher than that of stems (Figure 8).

### 3.6 Phylogenetic analysis of endophytic fungi of *Alpinia galanga*

**Figure 9:**
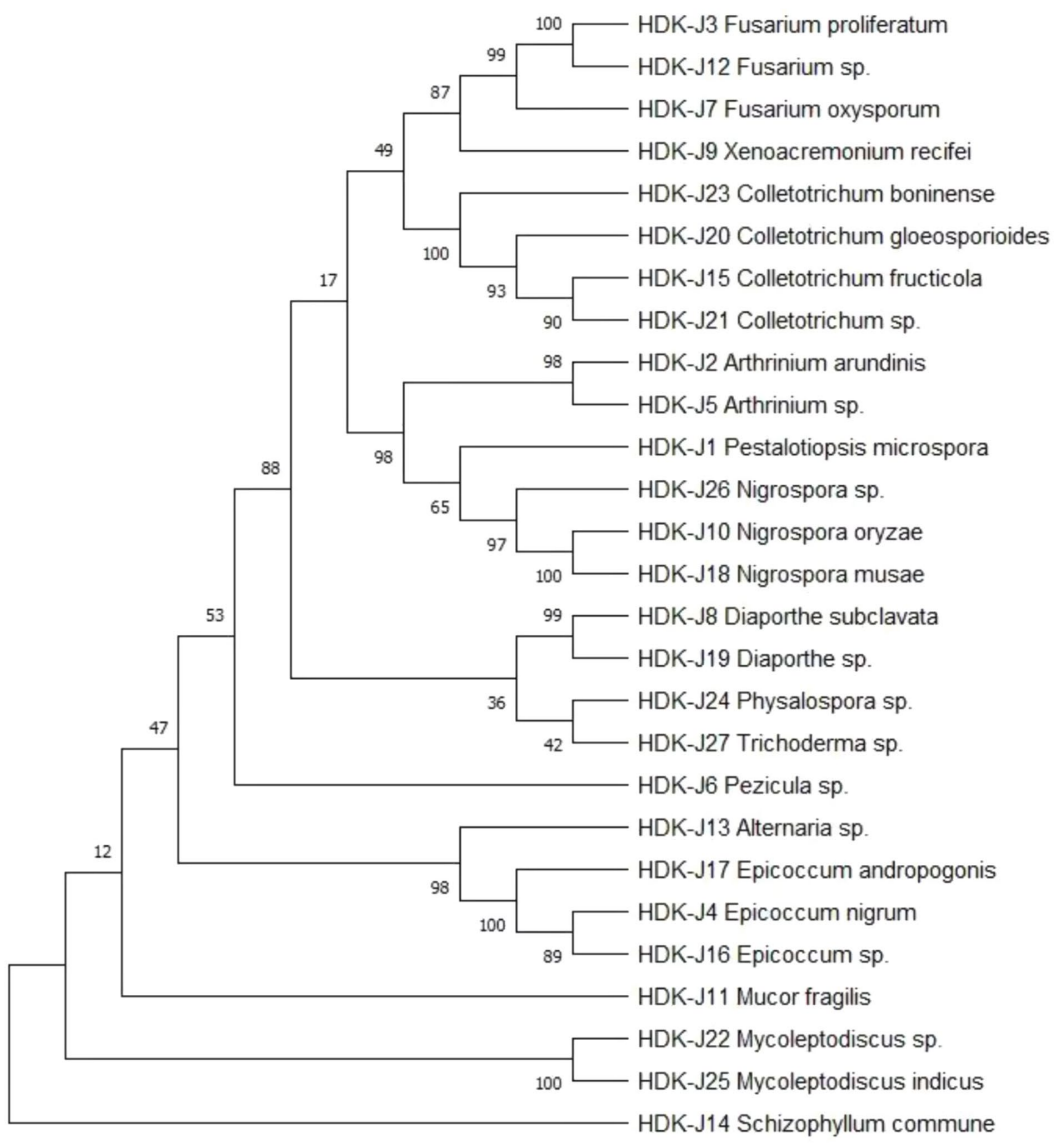
The phylogenetic relationships of endophytic fungi inferred from ML with MEGA in HDK_J.

**Figure 10:**
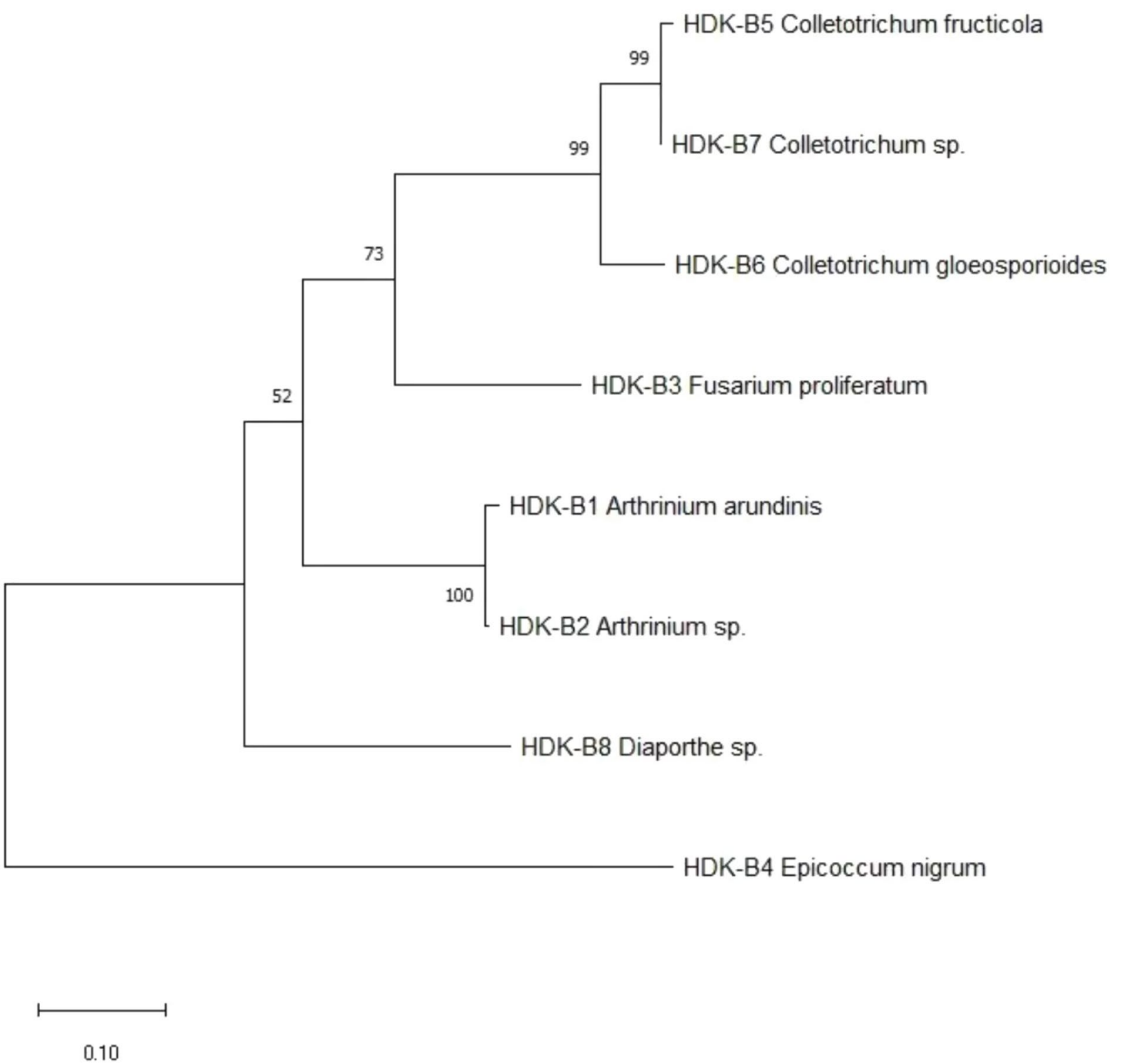
The phylogenetic relationships of endophytic fungi inferred from ML with MEGA in HDK_B.

The determined gene sequences of species with ≥98% similarity to the sequences downloaded from the NCBI database were edited into FASTA files using MEGA software, and the phylogenetic trees were constructed using the ML method (maximum likelihood method) with 1,000 bootstrap replications. The results indicated that the phylogenetic trees of endophytic fungi in HDK_J comprised four branches, with an average genetic similarity among genera of 52.80%. Of these, *S. commune* and *M. fragilis* belonged to Basidiomycota and Zygomycota, respectively, and other species belonged to Ascomycota. *Mycoleptodiscus* was listed as a sub-branch alone in Ascomycota; the others were merged into a sub-branch. The longer length of branches of *Ascomycota* indicated a more complete and more variable genetic relationship between species. In addition, different genera, such as *Fusarium* and *Xenoacremonium*, *Alternaria* and *Epicoccum*, and *Arthrinium* and *Pestalotiopsis*, were closely related to each other, whereas *Fusarium* was relatively distantly related to *Arthrinium*. At the same time, *Physalospora* and *Trichoderma*, although being in the same branch, had fewer connections with each other, with a support rate of only 42% (Figure 9). The phylogenetic tree for HDK_B had only one branch and was shorter, with an average genetic similarity between genera of 84.59%. In addition to the close relation between species within *Colletotrichum*, *Colletotrichum* was relatively closely related to *Fusarium* and was relatively distantly related to *Diaporthe* and *Epicoccum* (Figure 10).

## 4 Discussion and conclusion

The recruitment and community structure of endophytes in host plants are influenced by both external environmental factors and internal physiological states (Rodriguez et al., 2009; Brigham et al., 2023). External influences include biotic and abiotic factors, such as host diseases caused by bacterial infestation (Wang et al., 2023) and physiological pathologies caused by drought (Guevara-Araya et al., 2022), waterlogging (Ramakrishnan et al., 2024), soil salinization (Tang et al., 2023), atmospheric pollution (Meyer et al., 2010), heavy metal overload (Silva et al., 2024) and nutritional deficiencies (Yahya et al., 2023; Gao et al., 2024; Akram et al., 2023; Chaudhary et al., 2022; Redkar et al., 2022). The effects of these factors on host endophyte communities are complex and variable. In general, these external factors drive the change in targeted endophyte recruitment and the spatial selection of endophyte colonization by influencing the host’s internal metabolic activities (Morales-Vargas et al., 2024; Kumari et al., 2023; Lu et al., 2021). Healthy hosts usually form a relatively stable endophyte community, which assists the hosts in completing normal growth and development as well as material accumulation, and resists various abiotic factor stress and exogenous pathogen infection in the form of a single microbial species or functional microbial groups in the long-term host-endophyte interactions. Targeted recruitment of endophytes by the host is closely linked to the soil, rhizosphere, and leaf bulb microbial communities, and it has been shown that microbe-rich rhizosphere soils have higher diversity indices and endophyte abundance than microbe-deficient rhizosphere soils, which is particularly evident in perennial plants (Singh et al., 2017; Huang et al., 2023; Samuel et al., 2023). We found that endophytes were isolated from the tissue blocks of stems and leaves of Zingiberaceae plants that had been grown in pots using histocultured seedlings or rhizomes as asexual propagators, whereas samples from traditional planting areas with prolonged plantation had significantly higher diversity and abundance than those from non-traditional planting areas. Although the species and abundance of endophytes were closely associated with the microbial community attached to the body surface microenvironment of hosts, the community richness of endophytes was not consistent with that of other ecological niches. The species and abundance of endophytes change in different organs, with an overall decreasing trend: soil microorganisms > rhizosphere microorganisms > phyllosphere microorganisms > endophytes, with particularly pronounced changes in bacterial community (Guo et al., 2021; Pereira et al., 2023). It has been shown that host physiological activities and metabolite accumulation are closely related to the structure composition of endophyte community, and that differences in host metabolites can lead to significant changes in the structure of the endophyte community, especially in metabolite-rich medicinal plants (Ma et al., 2021; Caroline et al., 2023; Xi et al., 2024; Tang et al., 2023). Similarly, the numbers and species of endophyte populations will be reduced in the hosts of disease due to the reduction in the accumulation of substances or the changes of chemical composition, which are caused by reduced metabolic activities or the changes of normal metabolic pathways of hosts that are infected by pathogens (Wen et al., 2022; Vishwakarma et al., 2024; Jin et al., 2013). We found that the diversity and abundance of endophytic fungi in the stem and leaf tissues of diseased *A. galanga* were significantly lower than those of healthy *A. galanga*. At the species level, 27 endophytic fungi were isolated from healthy *A. galanga*, while eight endophytic fungi were isolated from diseased *A. galanga*. Further analysis revealed that among eight species of fungi isolated from both healthy and diseased hosts, five species of endophytic fungi, namely, *E. nigrum*, *C. gloeosporioides*, *C. fructicola*, *A. arundinis*, and *F. proliferatum*, belonged to hemibiotrophic fungi with a low degree of specialization, which are recruited to become endophytes in the non-host situation, and easily become pathogens after encountering the host, infecting the host, and causing disease. Meanwhile, the 27 endophytic fungi were generally fast-growing and metabolically vigorous, and some species had strong pigment-producing ability. It has been demonstrated that the recruitment and colonization and functional microbiome assembly of species are not random, but selective, and are controlled by host transmembrane pattern recognition receptors (PRRs), which exhibit host-active selective recruitment patterns called host-associated molecular patterns (MAMPs), and that any factor affecting the expression of PRR transmembrane proteins causes changes in species abundance and diversity (Saijo and Loo, 2020; Li T. R. et al., 2024). Recently, the PRR transmembrane proteins that interact with microorganisms have been found to be selective by knockout strategies, with different receptors recognizing different species (Olivieri et al., 2021; Alster et al., 2022; Li T. R. et al., 2024).

Plant endophytes combine with their hosts to form a holobiont, which assists the hosts in completing normal growth and development as well as in defense against external stresses. Thus, this holobiont, together with the microorganisms in the rhizosphere and the phloem layer, constitutes the plant microbiome, which characterizes the qualities of the hosts and maintains the health of hosts, and has the identity as “the second genome” of the hosts (Glick and Gamalero, 2021; Li X. F. et al., 2024). With the development of multi-omics analysis techniques, including genomics, transcriptomics, proteomics, and metabolomics, the function of the microbiome has begun to receive attention. The molecular mechanisms of the interactions between the host and the microbiome, as well as between different microbiomes, have been explored from the perspective of microecology in order to provide a more in-depth and comprehensive understanding of the functions and compositions of micro-ecosystems and even of the ecosystem as a whole (Chen et al., 2023; Zagal et al., 2024; Terna et al., 2024). In general, different microbiomes of ecological niche do not interact with the host independently, but form a complex rhizosphere microbiome-endophyte microbiome-phyllosphere microbiome interaction network mediated by the hosts, or two or three synergistically affecting the hosts and exercising the roles of functional microbiomes (Noguchi and Toju, 2024; Toju et al., 2018; Sun X. et al., 2023). In the plant–soil-microbial cycling ecosystem, soil is an important factor affecting the recruitment, assembly, and function of the plant microbiome. Bever et al. (2010) proposed the concept of “plant–soil feedback (PSF).” According to this theory, there are complex checks and balances between plants and soil, between soil pathogens and rhizosphere microbiomes, and between soil pathogens and host endophytes. If the positive feedback effect continues, soil-borne diseases will be contained, and on the contrary, if the negative feedback effect continues, the index of microbial diversity will decrease, leading to the reduction of community function, which has also been confirmed by our experimental results. We constructed the phylogenetic trees of endophytes of healthy and diseased *A. galanga* using the ML method from the perspective of culturable endophytes. We found that the phylogeny of endophytes of healthy stems and leaves of *A. galanga* had four major branches, belonging to Ascomycota, Basidiomycota and Zygomycota respectively, which were significant differences, and there were many longer sub-branches under the Ascomycota, suggesting that the genetic relationship on the branch of Ascomycota was more complete and the richer diversity. In contrast, there was only a branch of Ascomycota in the phylogenetic trees of the endophytic fungi in diseased stems and leaves of *A. galanga*. There were also a few sub-branches under Ascomycota, with close genetic relationships and small differences among genera, and a significant decrease in the diversity index, which was consistent with the study results of the sclerotinia rot disease (*Sclerotinia sclerotiorum*) by Zhang J. et al. (2024). Moreover, the host endophytic microbial community system includes not only the endophytic fungal community, but also the endophytic bacterial community and the endophytic actinobacterial community. It would be a meaningful study to explore how the synergistic effect among different communities affects host development by using multi-omics techniques, especially in the field of medicinal plants. With the discovery of more and more new active endophytic strains through genome sequencing, targeted construction of functional recombinant endophytic communities and artificially tailored synthetic microbial communities contribute to or promote host growth and development and component accumulation.

## Data Availability

The original contributions presented in the study are included in the article/supplementary material, further inquiries can be directed to the corresponding author.
